# A Randomized Double‐Blind Placebo‐Controlled First‐In‐Human Phase 1 Trial of TransCon PTH in Healthy Adults

**DOI:** 10.1002/jbmr.4016

**Published:** 2020-04-16

**Authors:** David B Karpf, Susanne Pihl, Sanchita Mourya, Eva Mortensen, Eshwari Kovoor, Denka Markova, Jonathan A Leff

**Affiliations:** ^1^ Ascendis Pharma, Inc. Palo Alto CA USA; ^2^ Ascendis Pharma A/S Hellerup Denmark

**Keywords:** CLINICAL TRIALS, HYPOPARATHYROIDISM, DISORDERS OF CALCIUM/PHOSPHATE METABOLISM, PTH, LONG ACTING PTH, THERAPEUTICS

## Abstract

TransCon PTH is a sustained‐release, essentially inactive prodrug transiently bound to an inert carrier, designed to release PTH(1‐34), and in development for hypoparathyroidism (HP). This phase 1, randomized, placebo‐controlled, single and multiple ascending dose (SAD and MAD, respectively) trial evaluated safety, tolerability, pharmacodynamics (PD), and pharmacokinetics (PK) of TransCon PTH in healthy adults. SAD and MAD cohorts consisted of 10 subjects (eight active, two placebo) who received up to seven single or six multiple ascending doses of TransCon PTH, respectively. TransCon PTH doses ranged from 3.5 to 124 μg PTH(1‐34) for the SAD cohorts and 3.5 to 24 μg PTH(1‐34)/day for the MAD cohorts. The primary PK endpoint was Free PTH. The PD endpoints included albumin adjusted serum calcium (sCa), fractional excretion of calcium (FECa), intact endogenous PTH(1‐84), bone turnover markers, renal tubular maximum reabsorption of phosphate/glomerular filtration rate (TMP/GFR), serum phosphate (sP) and magnesium, and 1,25 dihydroxyvitamin D. TransCon PTH was generally well tolerated; there were no drug‐related serious adverse events (SAEs), and all AEs were transient in nature. Free PTH demonstrated an effective half‐life of approximately 60 hours and a dose‐dependent, sustained exposure with an infusion‐like profile within the calculated physiologic range for active PTH at steady‐state. Albumin‐adjusted sCa demonstrated a dose‐dependent, sustained response with complete control of FECa despite modest hypercalcemia at higher doses. Renal tubular maximum reabsorption of phosphate/glomerular filtration rate (TMP/GFR) showed a dose‐dependent decrease, resulting in a dose‐dependent decrease in sP. TransCon PTH administered daily for 10 days showed no increase in the osteoblastic bone formation markers, serum bone‐specific alkaline phosphatase (BSAP) or P1NP, or the osteoclastic bone resorption marker, urine NTx, but modestly and transiently increased the osteoclast marker, serum CTx. These phase 1 data support TransCon PTH as a daily replacement therapy for HP providing physiological levels of PTH 24 hours per day and advancement into phase 2 clinical development. © 2020 The Authors. *Journal of Bone and Mineral Research* published by American Society for Bone and Mineral Research.

## Introduction

Parathyroid hormone (PTH) is a product of endocrine secretion of the four parathyroid glands located at or near the posterior capsule of the superior and inferior lobes of the thyroid gland. It is synthesized by chief cells as a prohormone peptide, which is initially 115 amino acids in length, then cleaved to 90, and eventually secreted as 84 amino acids; ie, PTH(1‐84). Although PTH(1‐84) is the biologically active form, receptor activation requires only the 31 to 34 amino acid N‐terminus fragment; ie, PTH(1‐31 to 1‐34).^1^ PTH regulates the body's extracellular calcium level within a very narrow range as well as phosphate homeostasis and bone turnover.

Hypoparathyroidism (HP) is a rare disease of impaired PTH production with an estimated number of affected patients in the United States of 60,000 to 115,000.^2^, ^3^ The majority of cases (75% to 78%) are acquired, occurring secondary to anterior neck surgery in which all four parathyroid gland(s) are inadvertently damaged or removed.^2^, ^3^ Among patients with chronic HP after total thyroidectomy, the risk of death over approximately a 4‐year follow‐up is twofold higher compared to patients without HP,^4^ although another smaller study in Denmark did not show an increase in mortality.^5^ In the setting of insufficient PTH secretion, renal reabsorption of filtered calcium is inappropriately low and reabsorption of phosphate is inappropriately high, contributing to hypocalcemia and hyperphosphatemia. In addition, conversion of 25‐hydroxyvitamin D to active vitamin D by the kidneys also lessens. Lack of active vitamin D leads to reduced calcium and phosphate absorption by the small intestines, and lack of PTH leads to decreased bone turnover. The net result is that patients with untreated HP develop hypocalcemia, hyperphosphatemia, an increased urinary fractional excretion of calcium (FECa) along with a more mineralized skeleton than age‐ and gender‐matched euparathyroid control subjects.

Standard‐of‐care (SoC) for chronic HP—specifically active vitamin D and calcium—only corrects hypocalcemia, targeting a serum calcium (sCa) just below or in the lower level of normal range to avoid worsening hypercalciuria, and is frequently associated with a sCa nadir prior to the next morning dose. SoC also does not restore PTH‐dependent renal calcium reabsorption, phosphate excretion, or normal bone turnover.^6^ In fact, long‐term active vitamin D and calcium may produce adverse effects beyond the original problems associated with HP, including an increase in calcium × phosphate product and urinary calcium (uCa) that together may lead to nephrocalcinosis, nephrolithiasis, and renal insufficiency as well as ectopic calcifications.^7^

Until recently, HP was the last remaining classic endocrine deficiency disorder for which the missing hormone was not available as a therapy.^2^ In 1996, Winer and colleagues^8^ established the experimental basis for using PTH(1‐34) to treat HP, showing that daily PTH(1‐34) injections are superior to SoC in treating adults and children with HP, allowing complete withdrawal of SoC while better controlling urinary calcium excretion. Follow‐up studies demonstrated that twice daily PTH(1‐34) injections are superior to SoC or once‐daily injections.^6^, ^8^, ^9^, ^10^, ^11^ Importantly, continuous subcutaneous (s.c.) PTH(1‐34) infusion via an insulin pump at a 62% to 65% lower daily dose, which produces physiological PTH levels over 24 hours, was superior to twice daily PTH(1‐34), resulting in more stable sCa levels, normal serum phosphate (sP), and serum magnesium (sMg) levels, normal uCa excretion, and a normalized rate of bone turnover in adults and children with HP while allowing complete withdrawal of SoC.^6^, ^12^ These studies demonstrated that renal tubules need more frequent PTH dosing than twice per day to optimally control uCa.^6^, ^13^ Despite currently available therapies, some patients still experience acute symptoms that can be severe and debilitating, negatively affecting activities of daily living and quality of life. Further, the long‐term consequences of hypercalciuria with subsequent renal failure is a major cause of morbidity among HP‐treated patients. Thus, a PTH replacement therapy that fully controls the disease, including episodes of hypercalcemia and hypocalcemia as well as hypercalciuria, remains an unmet need.

In 2015, rhPTH(1‐84) (Natpara; parathyroid hormone for injection) received US Food and Drug Administration (FDA) approval as an adjunct to calcium and active vitamin D to control hypocalcemia in patients with HP who are not well controlled on conventional therapy. Although the availability of once‐daily rhPTH(1‐84) has advanced the field, it does not provide PTH with a continuous pharmacokinetics (PK) profile that closely resembles the normal tonic secretory dynamics of PTH. The rhPTH(1‐84) label states that the incidence of hypocalcemic episodes did not decrease (while increasing the incidence of hypercalcemic episodes) and did not reduce or normalize uCa excretion.^14^ Therefore, an unmet need remains for a PTH replacement therapy that provides PTH with a continuous PK profile over 24 hours and more stably controls sCa and uCa levels.^13^, ^15^, ^16^

**Figure 1:**
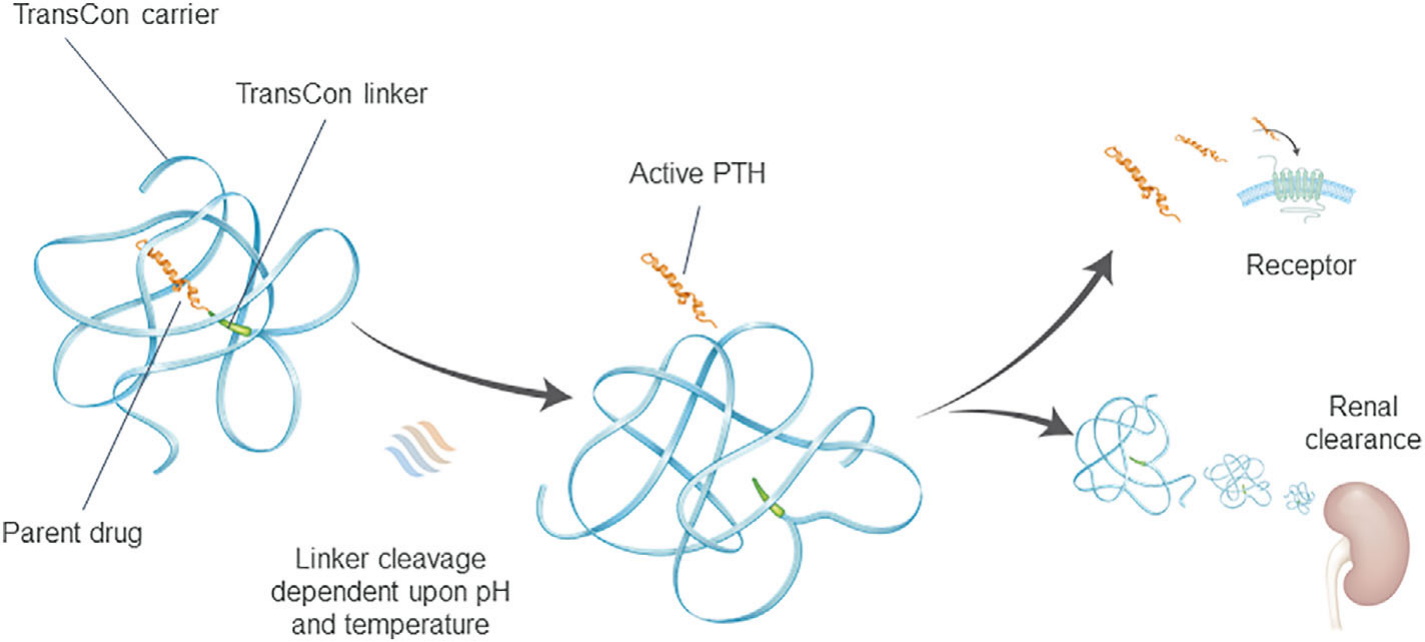
TransCon PTH, a long‐acting prodrug consisting of a parent drug, PTH(1‐34), transiently bound to a carrier via a linker that is autocleaved, releasing active PTH(1‐34) (and PTH(1‐33)).

TransCon PTH is a novel, long‐acting, essentially inactive prodrug designed to provide stable PTH levels in the physiological range for 24 hours per day, comparable to providing PTH(1‐34) by continuous s.c. infusion. Similar to other transiently conjugated compounds such as TransCon Growth Hormone,^17^ the prodrug consists of a parent drug, PTH(1‐34), transiently bound to an inert carrier (40 kDa mPEG) via a proprietary linker (Fig. 1). The carrier inactivates the parent drug and shields it from receptor uptake, renal clearance, and enzymatic degradation. Following a single s.c. injection and upon exposure to physiologic pH and temperature, autocleavage of the linker occurs, thus releasing active PTH in a controlled manner and leading to PTH exposure over several days.

The purpose of this trial was to evaluate the safety, tolerability, PK, and pharmacodynamics (PD) of single and multiple doses of TransCon PTH in healthy adults.

## Subjects and Methods

### Trial design

This was a phase 1, randomized, first‐in‐human, double‐blind, placebo‐controlled trial evaluating single and multiple ascending doses (SAD and MAD, respectively) of TransCon PTH. The trial was conducted at a single center, Nucleus Network, in Melbourne, Australia (Australian New Zealand Clinical Trials Registry ID ACTRN12617001375347). Prior to trial initiation, independent ethics committee approvals and informed consent from participants were obtained.

### Population

Healthy adult males and females between the ages of 18 and 60 years with a body mass index between 18.5 and 27 kg/m^2^ were eligible. Subjects with a history of malignancy, diabetes mellitus, PTH‐related or calcium‐related disorders, human immunodeficiency virus (HIV), hepatitis B or C, drug abuse, known hypersensitivity to components of the trial medication, or participation within 30 days in another trial were excluded. Pregnant or lactating females, subjects likely to be noncompliant, unable to complete the trial, or unable to follow the trial schedule were also excluded.

### Trial protocol

Subjects attended a screening visit; those who met eligibility criteria were randomly assigned to a SAD or MAD cohort (*n* = 10/cohort; eight active and two placebo) and received a single or multiple dose(s) of TransCon PTH, respectively, via a syringe and 31G 8‐mm needle s.c. to the abdomen. Single TransCon PTH doses ranged from 3.5 to 124 μg PTH(1‐34) (containing PTH(1‐34) [Bachem AG, Bubendorf, Switzerland], identical to the first 34 amino acids of rhPTH). Multiple TransCon PTH doses administered daily for 10 days ranged from 3.5 to 24 μg PTH(1‐34)/day. Throughout drug administration and for 4 days following the final dose, subjects remained housed for laboratory tests and safety monitoring.

### Safety assessments

At screening, demographic information, medical history, and concomitant medications, vital signs with height and weight, hematology, blood chemistry (including sCa, sP, and sMg), intact endogenous PTH(1‐84), 1,25‐dihydroxyvitamin D, and urine samples were collected. In addition, a limited physical examination and electrocardiogram (ECG) were performed.

Twice‐daily 12‐hour urine samples (including calcium, phosphorus, magnesium, and creatinine) were also collected. In MAD cohorts, serum samples collected from TransCon PTH‐treated and placebo‐treated subjects predose and postdose on day 14 were analyzed for anti‐PTH binding antibodies by BioAgilytix (Durham, NC, USA) using an electrochemiluminescence bridging assay. Samples were analyzed using a multitiered approach consisting of a screening assay and a confirmatory assay.

Via regular vital sign checks, physical examinations, and safety laboratory tests, subjects were also monitored for adverse events (AEs) throughout the trial, including injection site reactions (erythema, ecchymosis, edema, pruritus, and pain).

### PK and PD analyses

Because of long prodrug circulation time, some PTH(1‐34) is metabolized by a single amino acid into PTH(1‐33), which shows equipotency at the PTH/PTHrP receptor. The primary PK endpoints therefore included Free PTH (defined as the sum of Free PTH(1‐34) and Free PTH(1‐33)) as well as TransCon PTH.

The PD endpoints included albumin‐adjusted sCa, fractional excretion of calcium, sP, renal tubular maximum reabsorption of phosphate/glomerular filtration rate (TMP/GFR), intact endogenous PTH(1‐84), osteoblastic bone formation markers (serum bone‐specific alkaline phosphatase [BSAP] and P1NP), osteoclastic bone resorption markers (urine NTx and serum CTx), sMg, and 1,25‐dihydroxyvitamin D. PK samples were collected predose and at regular intervals postdose through 96 hours for the SAD cohorts and through day14 for the MAD cohorts.

The PK samples were analyzed by PRA Health Sciences (Assens, the Netherlands); Free PTH was quantified using protein precipitation, liquid‐liquid extraction, and solid‐phase extraction followed by ultra high‐performance liquid chromatography with tandem mass spectrometry detection measuring the two peptides, PTH(1‐34) and PTH(1‐33), by collection of both transitions simultaneously. Free PTH was calculated only if samples contained quantifiable levels of both Free PTH(1‐34) and Free PTH(1‐33); ie, above 1.40 and 0.958 pg/mL for Free PTH(1‐34) and Free PTH(1‐33), respectively. No lower limit of quantification was calculated for Free PTH because the ratio between Free PTH(1‐34) and Free PTH(1‐33) is not constant. Safety and PD samples were collected and analyzed by a local laboratory (SydPath Clinical Trials, Darlinghurst, Australia).

The PK parameters were calculated using Phoenix WinNonlin, version 6.4 (Certara USA, Inc., Princeton, NJ, USA). For Free PTH, t_max_ was defined as the time to maximum observed concentration and C_max_ was defined as the maximum observed concentration. Area under the curve (AUC) was calculated for the time from 0 to 24 and 96 hours postdose, respectively. Peak‐to‐trough was calculated as the ratio of C_max_ to C_24h_ at steady‐state. FECa was calculated based on time points when both serum and urine data were collected as follows:

(1) $$\text{FECa}=\left(\text{urinary calcium}\;x\;\text{serum creatinine}\right)/\left(\text{mean albumin}‐\text{adjusted serum calcium}\;x\;\text{urinary creatinine}\right)$$

The ratio, TMP/GFR is based on sP, urine phosphate (uP), serum creatinine (sCr), and urine creatinine (uCr) and was calculated as follows^18^:

(2) $$\mathrm{TMP}/\mathrm{GFR}=\mathrm{sP}–\left(\mathrm{uP}\;x\;\mathrm{sCr}/\mathrm{uCr}\right)$$

### Statistical analyses

All statistical analyses were performed using SAS 9.3 or higher (SAS Institute, Inc., Cary, NC, USA). Summary statistics were reported for continuous data, including mean, standard deviation, standard error, median, minimum, and maximum, whereas number and percentage of subjects within treatment groups were reported for categorical data. For sP and TMP/GFR, a linear regression model was performed for the change from baseline, with dose and baseline as independent variables at near steady‐state. Near steady‐state was defined as the average of predose values on day 8, 9, and 10. Dose was defined as a continuous variable where placebo was considered zero.

## Results

### Demographics

Overall, for the SAD cohorts, 56 subjects were randomized to TransCon PTH and 13 to placebo and received one dose of the study drug. Of these, 55 of 56 (98.2%) and 12 of 13 (92.3%) completed the trial, respectively. One subject administered TransCon PTH 32 μg PTH(1‐34) in SAD cohort 3 was withdrawn upon request for personal reasons 2 days early (day 3), whereas one subject administered placebo in SAD cohort 1 was withdrawn 1 day early (day 4) due to a serious adverse event (SAE), ie, bacteremia, assessed by the investigator to be unrelated to study drug.

For the MAD cohorts, 50 subjects were randomized to TransCon PTH and 13 to placebo and received at least one dose of the study drug. Of these, 46 of 50 (92.0%) and 10 of 13 (76.9%) completed the trial, respectively. Four of 50 (8.0%) administered TransCon PTH and 3 of 13 (23.1%) administered placebo withdrew from the trial early. Two of 50 (4.0%) administered TransCon PTH (MAD cohort 3) were replaced after being incorrectly dosed (see Safety), one of 50 (2.0%) withdrew upon request for personal reasons, and one of 50 (2.0%) withdrew due to a family emergency. Two of 13 (15.4%) administered placebo withdrew for personal reasons, and 1 of 13 (7.7%) experienced an SAE, ie, catheter site phlebitis, assessed by the investigator to be unrelated to the study drug. The demographic characteristics of the SAD and MAD cohorts were similar to placebo (Table 1).

**Table 1:** Demographic Characteristics of Trial Subjects (*n* = 132)

	SAD		MAD	
Characteristic	TransCon PTH (*n* = 56)	Placebo (*n* = 13)	TransCon PTH (*n* = 50)	Placebo (*n* = 13)
Age (years), mean ± SD	27.5 ± 9.2	28.6 ± 10.9	28.2 ± 6.6	25.0 ± 3.9
Male, *n* (%)	27 (48.2)	6 (46.2)	28 (56.0)	7 (53.8)
Race, *n* (%)				
White	53 (94.6)	12 (92.3)	42 (84.0)	13 (100.0)
Asian	2 (3.6)	1 (7.7)	6 (12.0)	0 (0)
Unknown	1 (1.8)	0 (0)	0 (0)	0 (0)
Multiple	0 (0)	0 (0)	2 (4.0)	0 (0)
Ethnicity, *n* (%)				
Hispanic/Latino	2 (3.6)	0 (0)	10 (20.0)	1 (7.7)
Non‐Hispanic/Latino	54 (96.4)	13 (100.0)	40 (80)	12 (92.3)
BMI (kg/m^2^), mean ± SD	22.7 ± 2.5	23.4 ± 2.0	23.1 ± 2.2	22.7 ± 2.5

BMI = body mass index; MAD = multiple ascending dose; SAD = single ascending dose; SD = standard deviation.

### Pharmacokinetics

**Figure 2:**
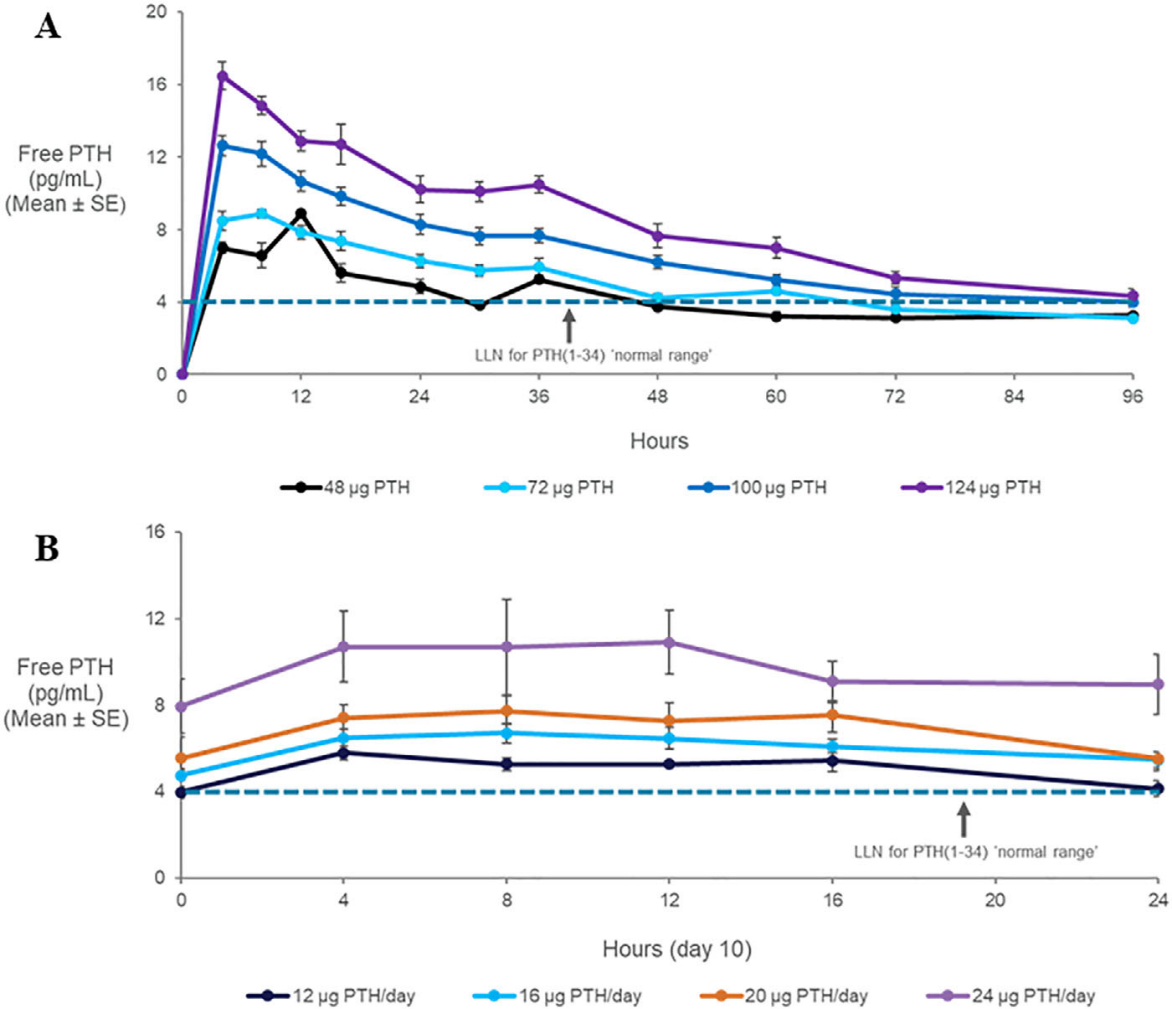
The mean ± SE plasma concentration of Free PTH (pg/mL) after (*A*) single or (*B*) multiple doses (at day 10) of TransCon PTH. Note, the calculated normal range for PTH(1‐34), 4 to 26 pg/mL, was derived from 40% of the molecular weight of PTH(1‐84). LLN = lower limit of normal.

Mean plasma concentrations of Free PTH following a single dose and daily dosings of TransCon PTH for 10 days are shown in Fig. 2. For the SAD cohorts, median time to peak Free PTH concentrations (t_max_) ranged from 4 to 8 hours postdose (with individual values ranging from 4 to 60 hours) and showed a dose‐dependent exposure over 96 hours. Plasma concentrations declined steadily after t_max_, with mean effective half‐life ranging from 51 to 56 hours across TransCon PTH 72 to 124 μg PTH(1‐34) (Fig. 2 *A*). For the MAD cohorts, median time to peak Free PTH concentrations (t_max_) on day 10 ranged from 4 to 8 hours postdose. The mean effective half‐life after multiple dosings was approximately 60 hours. On day 10, Free PTH also demonstrated a dose‐dependent response, with minimal decline in concentrations over the 24 hours following the tenth and final dose; the peak‐to‐trough ratios of Free PTH were consistent across the dose range, with mean values ranging from 1.3 to 1.6, indicating an infusion‐like profile. By day 8, Free PTH concentration in cohorts administered TransCon PTH 16 to 24 μg PTH(1‐34)/day appeared to be approaching steady state. (Samples were not analyzed for Free PTH concentrations in doses <12 μg PTH(1‐34)/day for day 1 to 8 because these concentrations were considered to be below the lower limit of quantification (Fig. 2 *B*).) From examination of the albumin‐adjusted sCa and Free PTH concentration versus time profiles, the sCa response correlated with the Free PTH level. The PK of Free PTH is summarized in Table 2.

**Table 2:** Summary of the Geometric Mean PK Parameters of Free PTH Following a Single s.c. Dose (SAD Cohorts) and 10 Daily s.c. Doses (MAD Cohorts)

Cohort	TransCon PTH dose	AUC_0‐t_ (h*pg/mL) mean (CV%)	C_max_ (pg/mL) mean (CV%)	t_max_ (h) median (min–max)	t_½_ (h) mean (CV%)	Peak‐to‐trough mean (CV%)
SAD	48 μg PTH(1‐34)	NC (*n* = 2)	6.61 (19.7) (*n* = 7)	8.0 (4.0–24.0) (*n* = 7)	NC (*n* = 2)	NA
	72 μg PTH(1‐34)	420 (20.3) (*n* = 5)	7.67 (28.5) (*n* = 7)	4.0 (4.0–60.3) (*n* = 7)	54.4 (18.9) (*n* = 5)	NA
	100 μg PTH(1‐34)	594 (17.3) (*n* = 8)	12.9 (11.7) (*n* = 8)	6.0 (4.0–8.0) (*n* = 8)	51.0 (32.4) (*n* = 7)	NA
	124 μg PTH(1‐34)	684 (36.9) (*n* = 8)	13.8 (45.5) (*n* = 8)	4.0 (4.0–60.0) (*n* = 8)	55.7 (37.1) (*n* = 7)	NA

	TransCon PTH dose	AUC_0‐24h_ (h*pg/mL) mean (CV%)	C_max_ (pg/mL) mean (CV%)	t_max_ (h) median (min–max)	t_½_ (h) mean (CV%)	Peak‐to‐trough mean (CV%)
MAD	12 μg PTH(1‐34)/day	122 (13.4) (*n* = 5)	6.24 (13.1) (*n* = 6)	4.0 (4.0–16.0) (*n* = 6)	64.8 (39.2) (*n* = 4)	1.55 (12.0) (*n* = 5)
	16 μg PTH(1‐34)/day	152 (9.4) (*n* = 5)	6.87 (16.8) (*n* = 7)	8.0 (4.0–12.0) (*n* = 7)	60.0 (20.3) (*n* = 4)	1.37 (6.0) (*n* = 5)
	20 μg PTH(1‐34)/day	165 (21.7) (*n* = 6)	8.30 (21.4) (*n* = 6)	6.1 (4.0–16.1) (*n* = 6)	51.7 (44.5) (*n* = 5)	1.54 (10.3) (*n* = 6)
	24 μg PTH(1‐34)/day	231 (24.2) (*n* = 3)	11.1 (27.6) (*n* = 3)	7.9 (4.0–12.0) (*n* = 3)	69.0 (22.3) (*n* = 3)	1.27 (4.4) (*n* = 3)

AUC = area under the curve; C_max_ = maximum observed concentration; CV = coefficient of variation; MAD = multiple ascending dose; NA = not applicable; NC = not calculated; SAD = single ascending dose; t_max_ = time to maximum observed concentration; t_1/2_ = half‐life.

### PD

**Figure 3:**
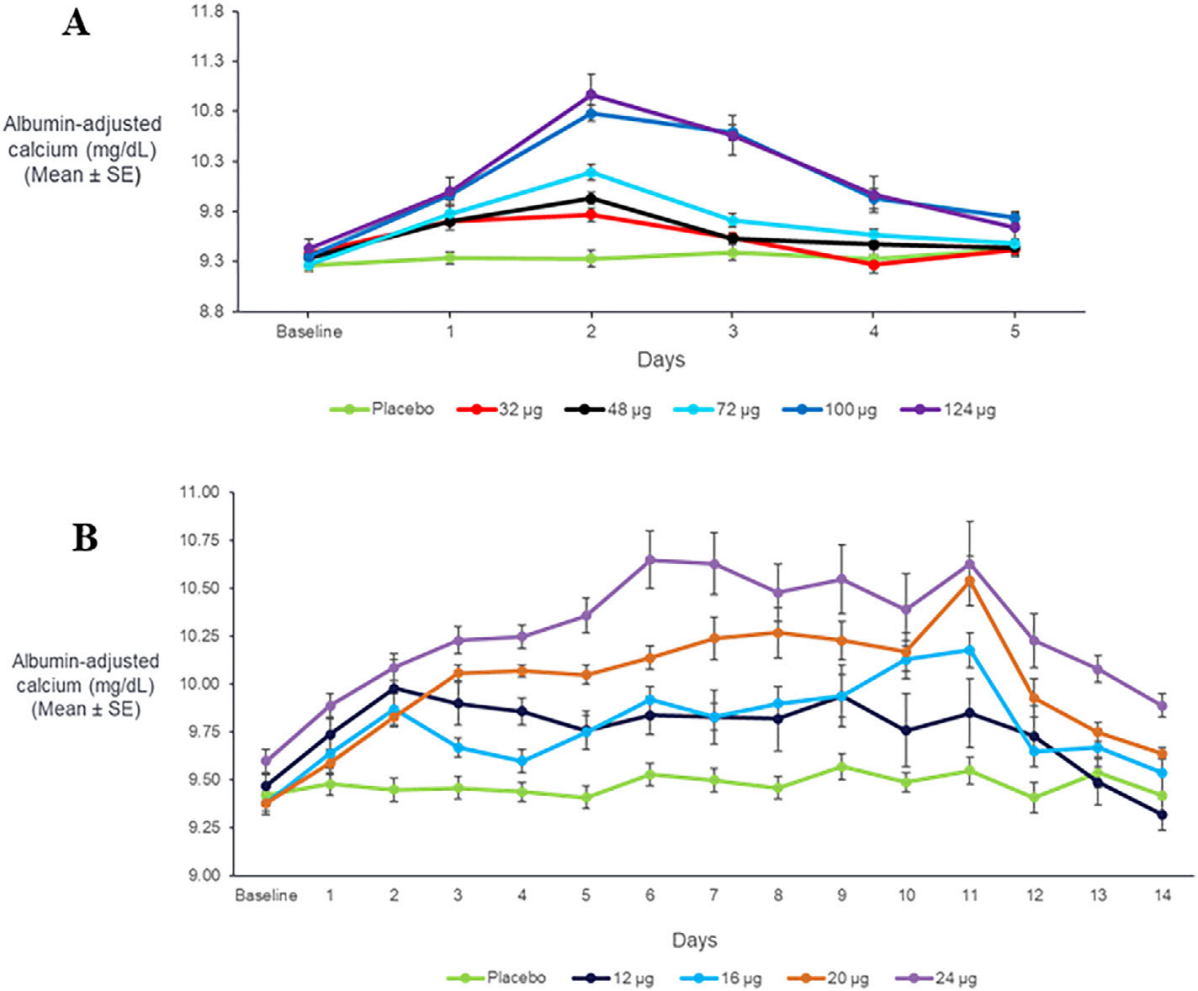
Mean ± SE change in plasma concentration of albumin‐adjusted serum calcium (mg/dL), following (*A*) a single dose or (*B*) daily doses for 10 days of TransCon PTH (*n* = 8/group). (Note: “Baseline” includes mean predose values on day 1 whereas “day 1” includes mean postdose values on day 1.) PTH = parathyroid hormone.

Initial increases in mean serum albumin‐adjusted sCa were seen in TransCon PTH 32 μg PTH(1‐34) SAD and TransCon PTH 12 μg PTH(1‐34)/day MAD cohorts, respectively, with SAD cohorts dosed with TransCon PTH ≥32 μg PTH(1‐34) and MAD cohorts administered TransCon PTH ≥12 μg PTH(1‐34)/day, showing a sustained dose‐dependent response (Fig. 3). The SAD cohorts demonstrated an increase in sCa for more than 3 days, with the maximal response on day 2, consistent with the approximately 60‐hour half‐life of Free PTH release from the prodrug (Fig. 3 *A*). In the MAD cohorts, stable dose‐dependent albumin‐adjusted calcium levels were seen after about 1 week of dosing, which did not return to baseline for 3 to 5 days after the final dose on day 10, consistent with the approximately 60‐hour half‐life for TransCon PTH (Fig. 3 *B*).

**Figure 4:**
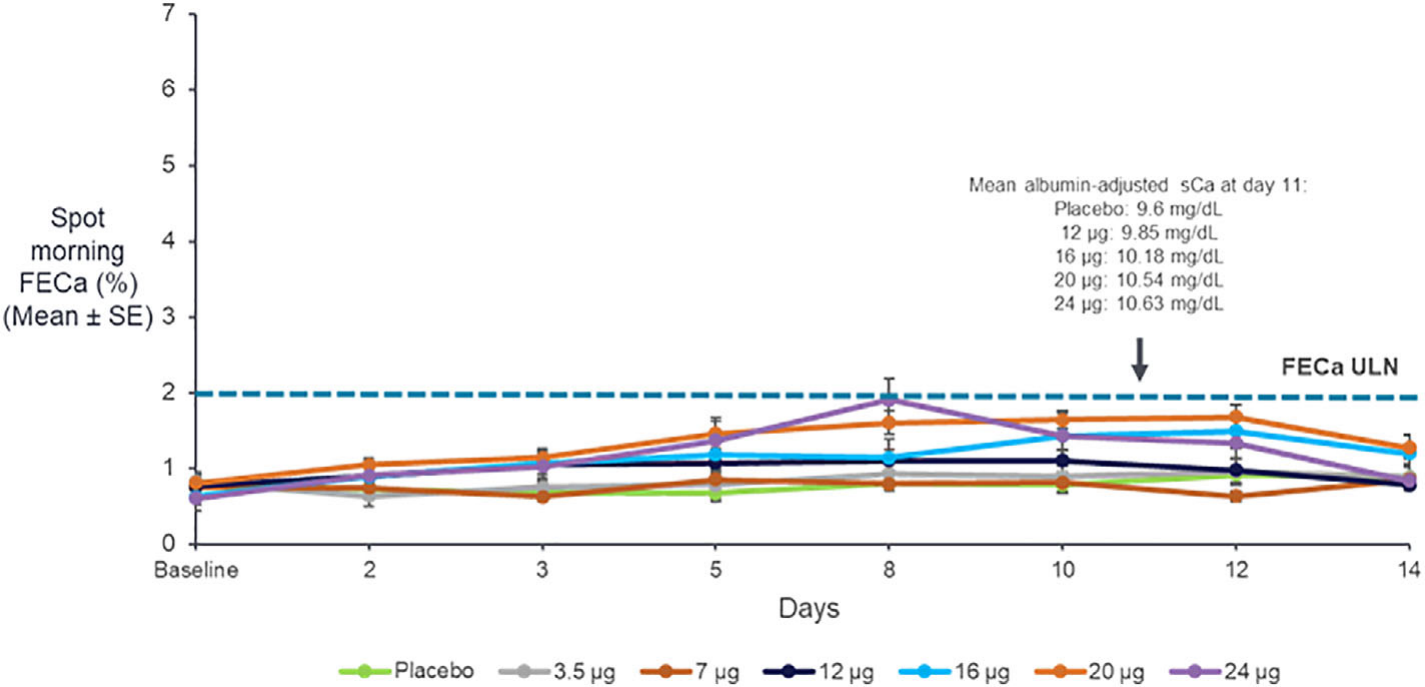
Spot FECa with daily doses of TransCon PTH (*n* = 8/group) for 10 days. ULN = upper limit of normal.

In the SAD TransCon PTH 124 μg PTH(1‐34) cohort (*n* = 8), mean ± SD albumin‐adjusted sCa increased from 9.43 ± 0.09 mg/dL at baseline to 10.97 ± 0.20 mg/dL on day 2. In the MAD TransCon PTH 24 μg PTH(1‐34)/day cohort (*n* = 8), adjusted sCa increased from 9.60 ± 0.06 mg/dL at baseline to 10.63 ± 0.22 mg/dL on day 11. However, despite mild hypercalcemia, FECa remained essentially within normal limits (approximately 0.4% to 2%) for both SAD (data not shown) and MAD cohorts (Fig. 4).

**Figure 5:**
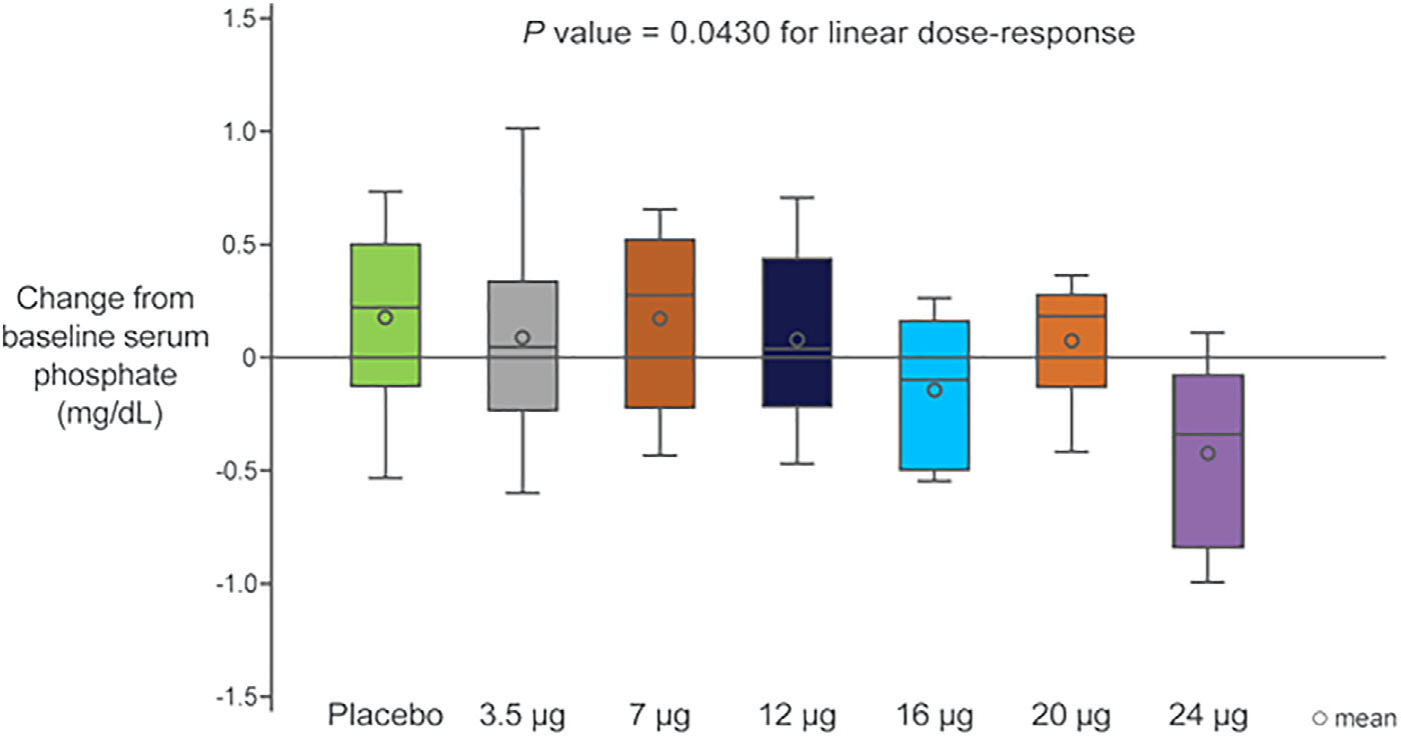
Box plots of the change of serum phosphate from baseline with daily doses of TransCon PTH for 10 days (values reflect the change from baseline over days 8 to 10).

**Figure 6:**
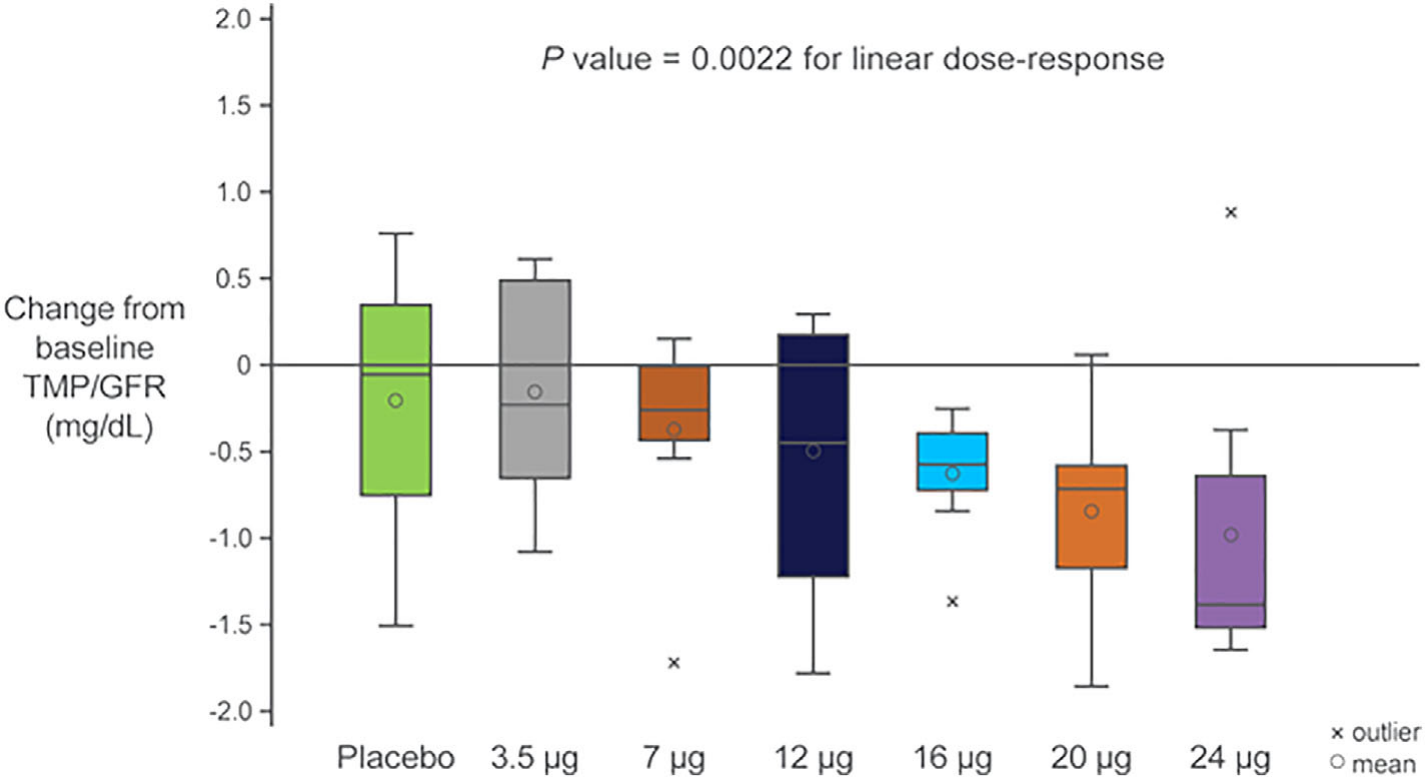
Phosphaturic effect with daily doses of TransCon PTH for 10 days (box plots reflect the change from baseline over days 8 to 10).

In the MAD cohorts, there was also a significant dose‐dependent decrease in sP (*p* = .0430) driven by a significant dose‐dependent decrease in TMP/GFR (*p* = .0022) (Figs. 5 and 6) and a nonsignificant increase in sMg (data not shown).

Of note, 1,25‐dihydroxyvitamin D levels did not change significantly in these normal healthy subjects (data not shown).

**Figure 7:**
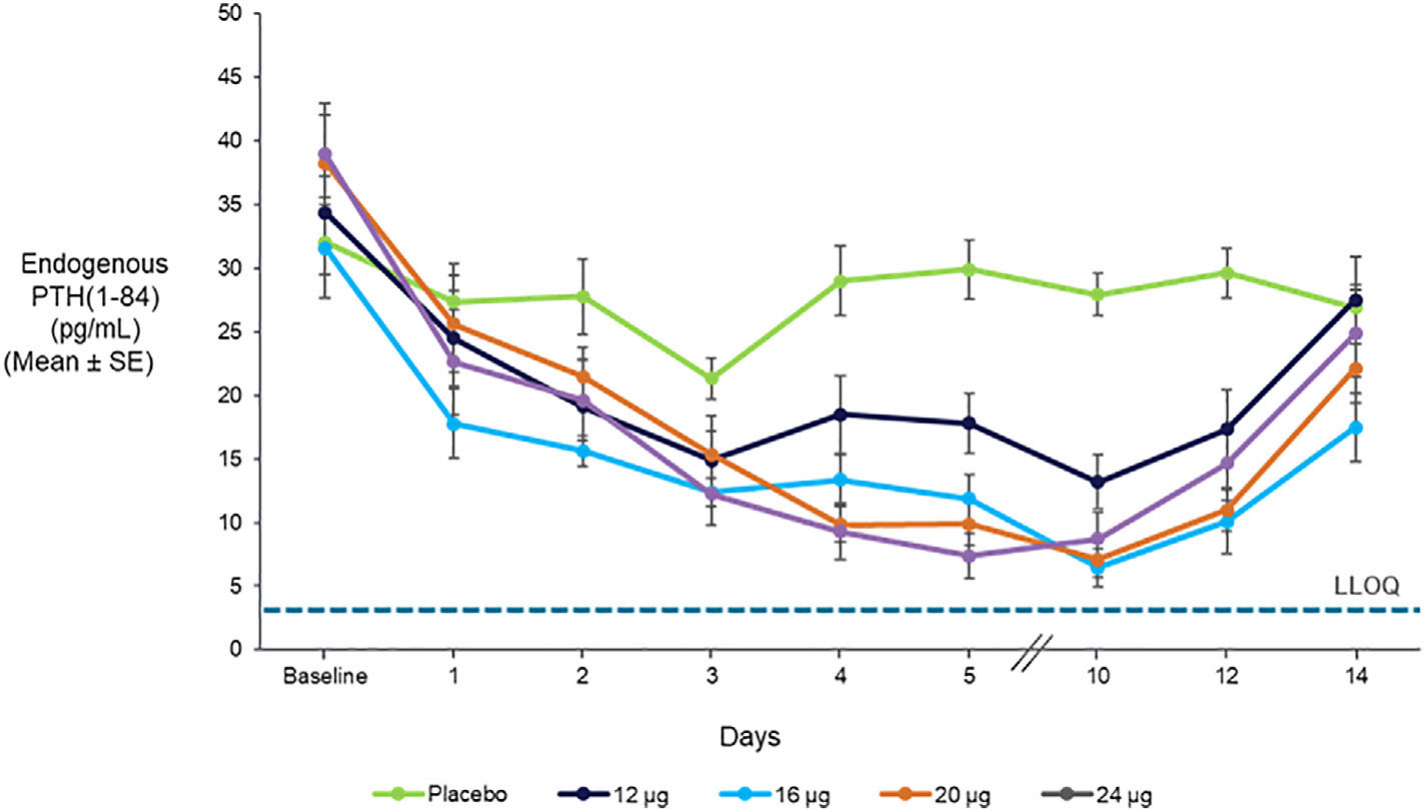
Suppression of intact endogenous PTH(1‐84) with daily doses of TransCon PTH (*n* = 8/group) for 10 days (the dashed line indicates the LLOQ; ie, 3 pg/mL). LLOQ = lower limit of quantification.

Intact endogenous PTH(1‐84) showed a dose‐dependent suppression in both the SAD cohorts (data not shown) and MAD cohorts (Fig. 7), as predicted by normal PTH physiology and the increase in sCa. Maximum suppression was achieved at a single dose of TransCon PTH ≥72 μg PTH(1‐34) and ≥16 μg PTH(1‐34)/day doses for the SAD and MAD cohorts, respectively.

**Figure 8:**
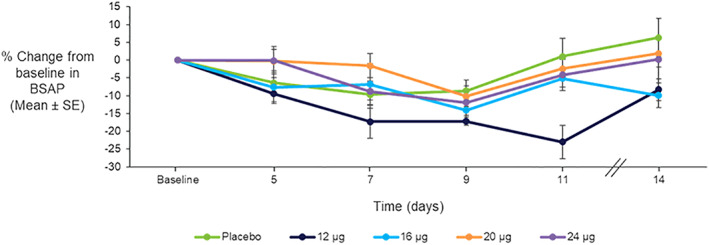
Mean ± SE percentage change from baseline in serum BSAP with daily doses of TransCon PTH (*n* = 8/group) for 10 days.

Compared to placebo, neither the osteoblastic bone formation marker, BSAP (Fig. 8) or P1NP (data not shown), increased over 10 days of TransCon PTH administration.

**Figure 9:**
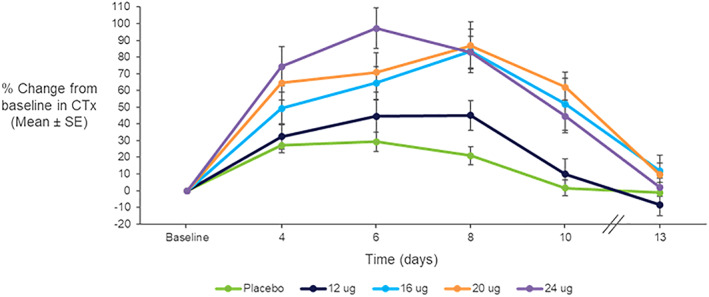
Mean ± SE percentage change from baseline in serum CTx with daily doses of TransCon PTH (*n* = 8/group) for 10 days.

For the osteoclastic bone resorption marker, urine NTx, no consistent increase was seen over 10 days (data not shown). The osteoclastic bone resorption marker, serum CTx, increased modestly over 10 days of TransCon PTH administration compared to placebo (Fig. 9). However, despite the fact that Free PTH levels were at steady‐state levels until at least day 11 (Fig. 2 *B*), CTx levels declined from day 8 to baseline by day 13.

### Safety

Only one subject in the SAD cohorts and one subject in the MAD cohorts, both of whom were administered placebo, discontinued the trial due to an AE. Both discontinuations were due to an SAE, ie, bacteremia and phlebitis, respectively. No subjects administered TransCon PTH discontinued the trial due to an AE.

In the SAD cohorts, 23 of 56 (41.1%) of subjects administered TransCon PTH and three of 13 (23.1%) administered placebo experienced at least one AE (Table 3). More subjects administered TransCon PTH 48 μg, 100 μg, and 124 μg PTH(1‐34) experienced at least one AE compared to the other treatment groups. In the SAD cohort administered TransCon PTH 12 μg PTH(1‐34), two subjects were mistakenly dosed with TransCon PTH 120 μg PTH(1‐34) due to pharmacy error; both were replaced. Two subjects experienced SAEs, one administered TransCon PTH 12 μg PTH(1‐34) developed catheter‐site phlebitis leading to hospitalization and one administered placebo developed bacteremia; neither SAE was assessed to be related to study drug and both resolved without sequelae.

**Table 3:** AEs by Cohort

Cohort	Dose	Size (*n*)	Any AE (%)	Any drug‐related AE (%)	Any moderate or severe AE (%)	Any SAE (%)
SAD	3.5 μg	8	1 (12.5)	1 (12.5)	0 (0)	0 (0)
	12 μg	8	3 (37.5)	1 (12.5)	1 (12.5)	1 (12.5)
	32 μg	8	3 (37.5)	2 (25.0)	0 (0)	0 (0)
	48 μg	8	6 (75.0)	5 (62.5)	2 (25.0)	0 (0)
	72 μg	8	2 (25.0)	0 (0.0)	1 (12.5)	0 (0)
	100 μg	8	4 (50.0)	3 (37.5)	1 (12.5)	0 (0)
	124 μg	8	4 (50.0)	2 (25.0)	1 (12.5)	0 (0)
	Total	56	23 (41.1)	14 (25.0)	6 (10.7)	1 (1.8)
	Placebo	13	3 (23.1)	1 (7.7)	3 (23.1)	1 (7.7)
MAD	3.5 μg	8	6 (75.0)	1 (12.5)	2 (25.0)	0 (0)
	7 μg	8	6 (75.0)	2 (25.0)	3 (37.5)	0 (0)
	12 μg	10	7 (70.0)	3 (30.0)	2 (20.0)	1 (10.0)
	16 μg	8	4 (50.0)	2 (25.0)	1 (12.5)	0 (0)
	20 μg	8	3 (37.5)	2 (25.0)	1 (12.5)	0 (0)
	24 μg	8	7 (87.5)	6 (75.0)	5 (62.5)	0 (0)
	Total	50	33 (66.0)	16 (32.0)	14 (28.0)	1 (2.0)
	Placebo	13	9 (69.2)	6 (46.2)	2 (15.4)	1 (7.7)

AE = adverse event; SAD = single ascending dose; SAE = serious adverse event; MAD = multiple ascending dose.

In the MAD cohorts, 33 of 50 (66%) administered TransCon PTH and nine of 13 (69.2%) administered placebo experienced at least one AE. More subjects administered TransCon PTH 24 μg PTH(1‐34)/day experienced at least one AE compared to the other treatment groups. Two subjects experienced SAEs, one administered TransCon PTH 12 μg PTH(1‐34)/day developed neutropenia in the setting of viral pharyngitis requiring brief hospitalization and one administered placebo developed catheter‐site phlebitis; neither SAE was assessed to be related to study drug and both resolved without sequelae.

The most common treatment‐emergent AEs in the SAD cohorts administered TransCon PTH were dizziness (10.7%), headache (7.1%), and postural dizziness (5.4%). The most frequently reported treatment‐emergent AEs for subjects who received placebo were headache, hypotension, bacteremia, and dysmenorrhea (all 7.7%). The most common treatment‐emergent AEs in the MAD cohorts administered TransCon PTH were headache (12%), dizziness (10%), palpitations and catheter site phlebitis (each 8%), postural dizziness and upper respiratory tract infections (each 6%), and presyncope and musculoskeletal pain (each 6%). The most frequently reported treatment‐emergent AEs for subjects who received placebo were headache, catheter site phlebitis, and catheter site pain (each 23.1%).

All potential vasodilatory AEs (ie, palpitations, orthostatic dizziness, orthostatic hypotension, postural dizziness, or syncope) adjudicated as possibly related to the study drug are shown in Table 4. A total of 18 subjects (14 subjects treated with TransCon PTH [13.2%] versus four subjects treated with placebo [15.4%]) experienced 21 potential vasodilatory AEs. Although these were more common in the highest MAD dosing cohort (TransCon PTH 24 μg PTH(1‐34)/day), subjects treated with placebo were at least as likely as subjects treated with TransCon PTH in this cohort to experience these AEs in this cohort. Specifically, vasodilatory or potentially vasodilatory AEs were reported by four of eight (50%) of subjects who were administered TransCon PTH and two of two (100%) who were administered placebo; all subjects who experienced vasodilatory symptoms were female.

**Table 4:** Vasodilatory Adverse Events by Cohort

Cohort	Dose	*n*	Tachycardia/palpitations (%)	Dizziness, light‐headedness (including postural/orthostatic dizziness, syncope, presyncope) (%)
SAD	3.5 μg	8	0 (0.0)	0 (0.0)
	12 μg	8	0 (0.0)	0 (0.0)
	32 μg	8	0 (0.0)	1 (12.5)
	48 μg	8	0 (0.0)	0 (0.0)
	72 μg	8	0 (0.0)	0 (0.0)
	100 μg	8	0 (0.0)	2 (25.0)
	124 μg	8	0 (0.0)	1 (12.5)
	Total	56	0 (0.0)	4 (7.1)
	Placebo	13	0 (0.0)	1 (7.7)
MAD	3.5 μg	8	1 (12.5)	1 (12.5)
	7 μg	8	0 (0.0)	1 (12.5)
	12 μg	10	0 (0.0)	1 (12.5)
	16 μg	8	0 (0.0)	0 (0.0)
	20 μg	8	1 (12.5)	0 (0.0)
	24 μg	8	3 (37.5)	2 (25.0)
	Total	50	5 (10.0)	5 (10.0)
	Placebo	13	1 (7.7)	2 (15.4)

A subject experiencing orthostatic dizziness and palpitations is counted twice in this table; all events were adjudicated as at least possibly related to study drug.

SAD = single ascending dose; MAD = multiple ascending dose.

One of three males in the TransCon PTH 24 μg PTH(1‐34)/day MAD cohort experienced symptomatic hypercalcemia (11.68 mg/dL) and discontinued dosing after the sixth dose; a second male subject was asymptomatic despite an albumin‐adjusted sCa of 11.76 mg/dL on day 11; none of the male subjects experienced vasodilatory AEs. As a result, TransCon PTH 20 μg PTH(1‐34)/day was identified as the maximum tolerable dose (MTD) in healthy volunteers. No MTD was observed in the SAD cohorts, even at the top dose of TransCon PTH 124 μg PTH(1‐34).

TransCon PTH was generally well tolerated. Injection site erythema (likely due to PTH‐related s.c. vasodilation) not associated with pain, itching, swelling, induration, or other symptoms was observed but was not captured as an AE and did not impact compliance with dosing.

No anti‐PTH antibodies were detected following s.c. administration of TransCon PTH. In addition, no safety concerns were detected on physical examination, by ECG, or in standard safety laboratory tests.

## Discussion

In this first‐in‐human trial, TransCon PTH was generally well tolerated; there were no drug‐related SAEs or severe AEs, and all AEs were transient in nature and comparable to placebo. Free PTH showed an effective half‐life of approximately 60 hours and a dose‐dependent, sustained exposure with an infusion‐like profile within the lower half of the physiologic range for active PTH at steady‐state. Similarly, the albumin‐adjusted sCa showed a dose‐dependent, sustained response that correlated with the Free PTH profile, with control of FECa despite mild hypercalcemia in the higher dose cohorts. TransCon PTH also showed the expected decrease in TMP/GFR and sP. TransCon PTH administered daily for 10 days showed no increase in the osteoblastic bone formation markers, serum BSAP or P1NP, or in the osteoclastic bone resorption marker, urine NTx, but did modestly and transiently increase the osteoclastic bone resorption marker, serum CTx.

Because of the short half‐life of calcitriol and currently available PTH‐based therapies, fluctuations in sCa, with mild hypocalcemia at trough and mild hypercalcemia at peak, may occur.^19^ These fluctuations may contribute to “brain fog,” impaired cognitive function reported by many patients with HP.^19^ National Institutes of Health (NIH)‐sponsored studies in adults and children with HP have shown that in terms of optimizing sCa, sP, sMg, and uCa, daily PTH(1‐34) injections are superior to SoC,^8^ twice‐daily PTH(1‐34) injections are superior to once‐daily injections,^9^, ^10^, ^11^ and continuous s.c. infusion of PTH(1‐34) is superior to twice‐daily PTH(1‐34) injections (while also normalizing the rate of bone turnover) despite more than a 60% lower dose of daily PTH.^6^, ^12^

The TransCon PTH PK and PD results mirror those of Winer and colleagues,^6^, ^12^ showing an infusion‐like profile at steady state with control of sCa, sP, and uCa and the absence of an anabolic effect on bone. Thus, once‐daily TransCon PTH may effectively treat patients with HP. Its prolonged half‐life of approximately 60 hours produced stable coverage of Free PTH within the lower half of the calculated normal range over 24 hours, which may provide greater stability of sCa and improve safety. The short half‐lives of both calcitriol and currently available PTH‐based therapies lead to daily sCa fluctuations, and stopping or missing a dose can lead to acute symptomatic and debilitating hypocalcemia.^14^ In contrast, due to its longer half‐life, daily TransCon PTH may lead to more stable sCa levels as well as a lower risk of severe hypocalcemia if a dose is missed. In the long‐term, improved control of sCa, sP, and uCa may also be associated with improved outcomes, allowing patients with HP to avoid the long‐term complications of chronic active vitamin D and calcium supplementation, including renal insufficiency, nephrolithiasis, and ectopic calcifications. Finally, normocalcemia obtained by an infusion‐like profile of PTH may allow complete discontinuation of active vitamin D and calcium, thus reducing overall pill burden and further contributing to improved quality of life for patients with HP.

Based on the approximate 60‐hour effective half‐life of Free PTH, eight to 12 daily doses of TransCon PTH are required to reach steady‐state, and accumulation was predicted. As a result, a single dose of TransCon PTH 32 μg PTH(1‐34) predicted the calcemic response of repeated TransCon PTH 12 μg PTH(1‐34)/day, and a single dose of TransCon PTH 100 μg PTH(1‐34) predicted the calcemic response of repeated TransCon PTH 20 to 24 μg PTH(1‐34)/day. The first MAD cohort to demonstrate an increase in sCa was administered TransCon PTH 12 μg PTH(1‐34)/day, which was associated with a Free PTH level of 5 pg/mL, in the lower end of the calculated normal range of PTH(1‐34). Higher doses of TransCon PTH resulted in dose‐dependent increases in both Free PTH and sCa, and as expected, suppression by negative feedback of intact endogenous PTH(1‐84) production, and all were associated with stable Free PTH levels within the lower half of the calculated normal range. These findings suggest the ability to individually titrate patients with HP into the normal calcemic range.

TransCon PTH may also positively impact hypercalciuria, a major cause of morbidity in patients with HP. In a study by Syed and colleagues,^20^ healthy volunteers were calcium‐clamped to sCa 10.3 mg/dL, which was associated with an increase in FECa to 6.0% to 7.0%. In contrast, when sCa was raised comparably by an infusion of PTHrP(1‐36), FECa did not differ from normal controls, showing the ability of PTHrP(1‐36) to stimulate renal calcium resorption.^20^ Notably, in both SAD and MAD cohorts administered TransCon PTH, in which higher doses produced an even higher sCa level, the FECa still remained normal (approximately 0.4% to 2%). These results reflect the continuous 24‐hour per day presence and activity of PTH released from the TransCon prodrug at the renal PTH/PTHrP receptor, leading to enhanced tubular reabsorption of calcium. The decrease in TMP/GFR and sP reflect the phosphaturic effect of PTH on the renal receptor.

In terms of bone turnover, SoC does not correct decreased osteoclast activity and diminished bone turnover associated with hypermineralized bone and increased bone mineral density (BMD) in HP. Because of their short half‐lives, currently available daily‐dosed PTH‐based therapies provide intermittent PTH exposure, stimulating osteoblastic bone formation as shown by increases in P1NP, BSAP, and trabecular BMD.^21^However, although anabolic activity resulting in increased BMD is the treatment goal in osteoporosis, it is not desirable in HP. The treatment objective is therefore to normalize bone turnover and modestly decrease BMD back to normal levels.

The TransCon PTH bone turnover data are similar to the published literature, which illustrate that continuous PTH administration is not anabolic. Winer and colleagues^6^, ^12^ demonstrated that continuous delivery of PTH(1‐34) restored bone turnover markers to normal levels compared to twice daily injections in both adults and children with HP but did not increase bone turnover markers above normal. Similarly, TransCon PTH showed no increase in the osteoblast markers, serum BSAP or P1NP. TransCon PTH did modestly and transiently increase the bone resorption marker, serum CTx, consistent with the data for continuous infusion of PTH(1‐34).^22^ However, the transient increase was substantially less than the persistent 200% increase in CTx previously observed with short‐acting PTH(1‐84).^21^ The absence of any increase in serum BSAP or P1NP contrasts with the significant increase in bone formation markers seen with currently available PTH‐based therapies^23^ and suggests that, by producing an infusion‐like profile of PTH within the calculated normal range, TransCon PTH is not anabolic. Future studies in patients with HP are needed to determine if treatment beyond 10 days leads to normalization of bone turnover and a normalization (ie, a modest decline) in the elevated BMD observed in patients with HP treated with SoC and are being evaluated in a phase 2 trial.

In the subjects who received TransCon PTH 24 μg PTH(1‐34)/day, AEs reflected the known pharmacology of PTH, ie, vasodilatation (as well as hypercalcemia), leading to orthostatic hypotension, lightheadedness, tachycardia, and syncope; similar AEs were seen in the placebo‐treated subjects in this cohort as well. Vasodilatory symptoms such as these have been seen previously with PTH‐based therapies.^24^ These symptoms are usually transient, present only for the initial few weeks of treatment, and can be managed by administering the drug at bedtime. Because participants in this trial were all healthy volunteers with normal baseline calcium levels, where even a 1.5 mg/dL increase in sCa made them hypercalcemic, it is possible that higher doses, ie, greater than TransCon PTH 20 μg PTH(1‐34)/day may be well tolerated and effective in patients with HP.

This trial had limitations. Although the trial was blinded, TransCon PTH was designed to increase PTH and therefore sCa levels; in later SAD and MAD cohorts administered higher doses, rises in sCa may have made it obvious to investigators which subjects were randomized to TransCon PTH versus placebo. As a phase 1 study, only healthy volunteers were included. Although calcium metabolism should not differ between populations, care should be exercised in extrapolating results to patients with HP who have underlying hormone and electrolyte abnormalities at baseline. No anti‐PTH binding antibodies were detected. However, assessments were made only 14 days after the first of 10 doses of TransCon PTH. Immunogenicity will be assessed with longer‐term dosing in future clinical trials. Finally, the trial was not blinded related to treatment dose; overreporting of side effects associated with the study drug may have occurred, especially in the higher‐dose cohorts.

TransCon PTH demonstrated potent calcemic, renal calcium reabsorption, and potent phosphaturic effects, indicating control of sCa and phosphate as well as uCa, and no evidence of any anabolic effect. The results of this phase 1 trial support TransCon PTH as a replacement therapy for HP, providing physiological levels of PTH 24 hours per day, and advancement into phase 2 clinical development.

## Disclosures

DBK, EM, EK, SM, and DM are employees of Ascendis Pharma, Inc.; JAL is a former employee of Ascendis Pharma, Inc. SP is an employee of Ascendis Pharma A/S.
